# Chronic Kidney Disease: Interaction of Adiponectin Gene Polymorphisms and Diabetes

**DOI:** 10.3390/ijms24098128

**Published:** 2023-05-01

**Authors:** Hsi-Hsien Chen, Ya-Li Huang, Mei-Chieh Chen, Chih-Yin Wu, Ying-Chin Lin, Horng-Sheng Shiue, Sheng-Lun Hsu, Yu-Mei Hsueh

**Affiliations:** 1 Division of Nephrology, Department of Internal Medicine, School of Medicine, College of Medicine, Taipei Medical University, Taipei 110, Taiwan; 570713@yahoo.com.tw; 2 Division of Nephrology, Department of Internal Medicine, Taipei Medical University Hospital, Taipei 110, Taiwan; 3 Department of Public Health, School of Medicine, College of Medicine, Taipei Medical University, Taipei 110, Taiwan; 4 Department of Microbiology and Immunology, School of Medicine, College of Medicine, Taipei Medical University, Taipei 110, Taiwan; mcchen@tmu.edu.tw; 5 Graduate Institute of Medical Sciences, College of Medicine, Taipei Medical University, Taipei 110, Taiwan; 6 Department of Family Medicine, Wan Fang Hospital, Taipei Medical University, Taipei 110, Taiwangreen1990@tmu.edu.tw (Y.-C.L.);; 7 Department of Family Medicine, School of Medicine, College of Medicine, Taipei Medical University, Taipei 110, Taiwan; 8 Department of Occupational Medicine, Wan Fang Hospital, Taipei Medical University, Taipei 110, Taiwan; 9 Department of Chinese Medicine, College of Medicine, Chang Gung University, Taoyuan 333, Taiwan

**Keywords:** adiponectin, polymorphisms, diabetes, chronic kidney disease, arsenic, cadmium

## Abstract

Adiponectin is an adipokine multipeptide hormone with insulin-sensitizing; anti-atherosclerotic; and anti-inflammatory properties. Chronic kidney disease (CKD) may be associated with low adiponectin. The adiponectin gene *ADIPOQ* is thought to be the only major gene responsible for plasma adiponectin levels; which are associated with diabetes and diabetic nephropathy. The purpose of this study was to investigate the association between *ADIPOQ* polymorphism and CKD. In addition; the combined effects of *ADIPOQ* polymorphism and diabetes and levels of total urinary arsenic and blood cadmium on CKD were also explored. This study included 215 CKD patients and 423 age–sex matched controls. The *ADIPOQ* polymorphisms were determined using the Agena Bioscience Mass ARRAY System. The levels of blood cadmium and urinary arsenic species were measured. The *ADIPOQ* rs182052 GA/AA genotype had a marginally lower odds ratio (OR) for CKD than the GG genotype. The OR (95% confidence interval; CI) was 16.33 (5.72–46.66) of CKD in subjects carrying the *ADIPOQ* rs182052 GG genotype and diabetes compared to non-diabetes subjects carrying the *ADIPOQ* rs182052 GA/AA genotype; the interaction term had *p* = 0.015; and the synergy index was 6.64 (1.81–24.36) after multivariate adjustment. A significant interaction of diabetes and *ADIPOQ* rs1501299 risk genotype increased the OR of CKD after multivariate adjustment with a synergy index of 0.31 (0.11–0.86) and a multiplicative interaction with *p* = 0.001. These results suggest that *ADIPOQ* rs182052 and rs1501299 risk genotypes may significantly modify the association between diabetes and CKD but not the association between total urinary arsenic and blood cadmium and CKD.

## 1. Introduction

Chronic kidney disease (CKD) is a growing public health problem. The prevalence of CKD in Taiwan was 11.9%, defined as an estimated glomerular filtration rate (eGFR) of <60 mL/min/1.73 m^2^ [1]. According to the Global Burden of Diagnosis analysis in 2017, the global prevalence of CKD was 9.1% (approximately 697.5 million cases), an increase of 29.3% since 1990 [2]. Taiwan has the highest prevalence of end-stage renal disease (ESRD) in the world, and ESRD incidence increased steadily from 2010 to 2018 [3]. Therefore, it is very important for Taiwan to explore CKD etiology.

Type 1 diabetes, type 2 diabetes, glomerulonephritis, and hypertension are considered risk factors for CKD [2]. A review study showed that exposure to environmental pollutants, such as heavy metals, is also a risk factor for CKD [4]. Our previous study found that high levels of blood cadmium and lead and total urinary arsenic significantly decreased eGFR and increased odds ratios (ORs) for CKD, whereas plasma selenium concentrations significantly increased eGFR and decreased OR for CKD [5]. However, the mechanisms by which metal or metalloid concentrations in blood or urine are associated with CKD are not fully understood.

Adiponectin is a 30-kDa protein that controls glucose and fatty acid metabolism, regulates insulin sensitivity, inhibits the proliferation of vascular smooth muscle cells, has general anti-inflammatory effects, and regulates atherogenesis [6,7,8]. The reduction of adiponectin leads to insulin resistance [8], which may be related to an increased risk of diabetes [9]. Plasma adiponectin levels depend on renal function and are significantly elevated in patients with renal insufficiency [10]. Plasma adiponectin levels in pre-dialysis CKD patients were negatively correlated with glomerular filtration rate [11,12]. However, a two-sample bidirectional Mendelian randomization study noted that high adiponectin concentration can increase eGFR levels and reduce CKD risk [13]. The serum adiponectin level may be able to improve eGFR and treat CKD caused by decreased eGFR [13]. The association between adiponectin levels and CKD is inconsistent.

Exposure to inorganic arsenic from drinking water decreased serum adiponectin and interfered with lipid metabolism in mice [14]. Another animal experiment also found that arsenic exposure reduces adiponectin and reduces body weight [15]. Long-term exposure to low-dose cadmium in rats downregulated adiponectin and leptin mRNA expression levels, suggesting a toxic effect of cadmium on adipocyte function [16]. Expression levels of adiponectin mRNA in white adipose tissue were reduced in mice exposed to high doses of cadmium for 6 h or 2 weeks, suggesting that cadmium exposure may lead to the development of insulin resistance, hypertension, and cardiovascular disease [17]. Results from the National Study of Women’s Health showed that cadmium and lead in urine samples were associated with adverse levels of high molecular weight adiponectin [18]. Experiments in mice showed that selenium supplementation can increase adiponectin concentration [19], but randomized clinical trials showed that selenium supplementation is not associated with adiponectin concentration [20]. The relationships between arsenic, lead, cadmium, and selenium exposures and adiponectin remain inconsistent.

Adiponectin is encoded by the *ADIPOQ* gene, which consists of two introns and three exons located on chromosome 3q27 [21]. Genetic variation in the adiponectin-encoding gene was associated with adiponectin levels in a genome-wide linkage and association study [22]. Various *ADIPOQ* polymorphisms were associated with coronary artery disease [23], diabetes [24], and hypertension [25]. Currently, only one study has examined the distribution of the *ADIPOQ* rs1501299 TT genotype, which significantly differed between CKD and controls, but without adjusting for other confounding factors [26]. Therefore, the purpose of this study is to investigate the association between *ADIPOQ* polymorphisms and CKD. In addition, we also assessed whether the *ADIPOQ* genotype modifies the associations between a history of diabetes and hypertension, total urinary arsenic and blood cadmium concentrations, and CKD.

## 2. Results

The sociodemographic characteristics, lifestyle, and disease history for CKD cases and controls are shown in Table 1. The OR of CKD was significantly lower in those with an education level above high school and who drink alcohol, tea, or coffee frequently or occasionally than in those with an education level below high school and who never drink alcohol, tea, or coffee. The proportion of smokers did not differ between cases and controls. Routine use of analgesics had a significantly higher OR for CKD than those who used analgesics only when needed. Individuals with diabetes and hypertension had a significantly higher OR for CKD than those without.

The association between *ADIPOQ* genotypes and CKD is presented in Table 2. The *ADIPOQ* rs1501299 GT genotype had a marginally significantly higher OR for CKD than the GG genotype. After multivariate adjustment, the OR of CKD was marginally higher in those with the *ADIPOQ* rs182052 GG genotype compared to the GA/AA genotype. While the *ADIPOQ* rs1501299 GT and *ADIPOQ* rs182052 GG genotypes show marginal values. If the results of *ADIPOQ* genotyping improve the health of CKD, it is worthwhile [27]. The other polymorphisms of *ADIPOQ* were not associated with CKD.

The LD and haplotype analyses showed that *ADIPOQ* had two haplotype blocks (Supplementary Figure S1). The Lewontin’s D′ for the haplotype *ADIPOQ* block 1 (*ADIPOQ* rs182052 and rs822396) and *ADIPOQ* block 2 (*ADIPOQ* rs1063539 and rs1501299) polymorphisms was 0.96 for both, indicating the LD (Figure S1A) and r^2^ values (Figure S1B) for each pair of polymorphisms. Supplementary Table S2 shows the relationship between the *ADIPOQ* haplotypes and CKD. The OR of CKD in the A-A haplotype of *ADIPOQ* block 1 marginally decreased compared to the G-A haplotype, and the haplotypes in *ADIPOQ* block 2 were not related to CKD after multivariate adjustment.

Table 3 shows the serum creatinine, plasma selenium, blood cadmium and lead, and total urinary arsenic concentrations and eGFR in the CKD cases and controls. Of these, plasma selenium levels and eGFR were significantly lower in CKD cases than controls, but blood cadmium and lead, and total urinary arsenic concentrations were significantly higher; these results are similar to previous studies [5,28].

The association between *ADIPOQ* polymorphisms and CKD was analyzed after stratification by medical history, such as diabetes and hypertension, blood cadmium and lead, plasma selenium, and total urinary arsenic (Table 4). The multivariate-adjusted OR of CKD in diabetic patients with the *ADIPOQ* rs182052 GG genotype was significantly higher than those with the GA+AA genotype, while the *ADIPOQ* rs182052 genotype was not associated with CKD in non-diabetic patients. When comparing the *ADIPOQ* rs182052 GG with the GA+AA genotype, the OR of CKD was marginally higher in the low total urinary arsenic and low blood cadmium concentration group but not in the high total urinary arsenic or high blood cadmium concentration group. Diabetics with *ADIPOQ* rs1501299 GT/TT versus GG genotype had significantly lower multivariate-adjusted OR of CKD. In contrast, the multivariate-adjusted OR of CKD in the *ADIPOQ* rs1501299 GT/TT genotype without diabetes was marginally higher than those with GG genotype. Thus, *ADIPOQ* rs182052 may, respectively, interact with diabetes and total urinary arsenic and blood cadmium concentrations on CKD. *ADIPOQ* rs1501299 may interact with diabetes on CKD. Therefore, we subsequently analyzed the interaction of these *ADIPOQ* genotypes and diabetes and blood cadmium and total urinary arsenic concentrations on CKD. However, there was no association between *ADIPOQ* polymorphisms and CKD in analyses stratified by hypertension, blood lead, and plasma selenium.

Table 5 shows pairwise analyses of the interaction of diabetes, total urinary arsenic, blood cadmium, and *ADIPOQ* risk genotype on CKD. The OR for CKD increased significantly with increasing risk factors in a dose–response manner. The OR (95% confidence interval, CI) of CKD was 16.33 (5.72–46.66) in subjects carrying the *ADIPOQ* rs182052 GG genotype with diabetes compared to those with the *ADIPOQ* rs182052 GA/AA genotype and without diabetes. The *ADIPOQ* rs182052 and diabetes status significantly interacted with CKD (*p* = 0.015), and the synergy index was 6.64 (1.81–24.36). Thus, *ADIPOQ* rs182052 appeared to have an additive and multiplicative interaction with diabetes on CKD after multivariate adjustment. The OR of CKD increased significantly with a risk factors (diabetes and *ADIPOQ* rs1501299 risk genotype) increment after multivariable adjustment, with an additive interaction (synergy index) of 0.31 (0.11–0.86) and a significant multiplicative interaction (*p* = 0.001). The interaction between the *ADIPOQ* rs182052 risk genotype and metals showed a dose-dependent OR for CKD when increasing risk factors, but the interaction was not significant.

## 3. Discussion

We found the OR of CKD in the *ADIPOQ* rs182052 GG genotype was marginally higher than that of the GA/AA genotype. There was no evidence for an association of other *ADIPOQ* genotypes or haplotypes with CKD in this study (Table 2). However, stratification analysis showed that *ADIPOQ* rs1501299 and rs182052 had different ORs of CKD in different strata of diabetes and levels of blood cadmium and total urinary arsenic (Table 4). The combined effect of *ADIPOQ* rs1501299 or *ADIPOQ* rs182052 risk genotypes and diabetes, respectively, increased the OR of CKD when risk factors rose in a dose–response manner and had significant additive and multiplicative interaction effects (Table 5).

Our study found that the OR of CKD was significantly lower in those who drank alcohol, tea, and coffee (Table 1). This may be because alcohol [29] and tea [30] contain polyphenols that can reduce reactive oxygen species and anti-inflammatory components, and coffee contains active phytochemicals, including polyphenols, that can increase antioxidant capacity [31] and modulate glucose and fat metabolism [32].

The *ADIPOQ* rs1501299 polymorphism is in intron 2 at position 276 of the adiponectin gene (SNP 276G>T). In a Spanish study, the *ADIPOQ* rs1501299 T allele increased plasma adiponectin levels and decreased insulin resistance [33]. Another study showed that the *ADIPOQ* rs1501299 T allele was associated with the pathogenesis of type 2 diabetes in Kyrgyzstan [34]. In a renal transplant study, the T allele of *ADIPOQ* rs1501299 in donors was significantly associated with higher eGFR values than the G allele and was also associated with higher combined genetic scores and better functions in a Spanish study [35]. The inconsistent results may be attributed to ethnic differences in the distribution of the *ADIPOQ* rs1501299 polymorphism. However, our study showed that the *ADIPOQ* rs1501299 GT/TT genotype significantly decreased the OR for CKD compared with the GG genotype in subjects with diabetes (Table 4). This is because the *ADIPOQ* rs1501299 TT genotype increased the plasma adiponectin concentration, reduced the insulin resistance index, and decreased the risk of type 2 diabetes compared to the GG genotype in a Japanese population [36], thereby reducing the risk of CKD. Conversely, *ADIPOQ* rs1501299 GT/TT relative to the GG genotype increased the OR of CKD in subjects without diabetes in our study (Table 4). This result is consistent with a Taiwanese study showing that the *ADIPOQ* rs1501299 T allele increased the risk of type 2 diabetes [37]. A Greek study showed that the OR of the *ADIPOQ* rs1501299 GT genotype significantly increased the risk of diabetic retinopathy in diabetic patients, and the OR (95% CI) was 2.68 (1.38–5.21) when adjusted for other risk factors [38]. A meta-analysis study found that the *ADIPOQ* rs1501299 GG genotype had a marginally lower OR for coronary artery disease than the GT/TT genotype in East Asians with an OR of 0.82 and 95% CI 0.66–1.03 [39]. These inconsistent results may be due to complex interactions between genotype and environmental factors (such as the presence or absence of diabetes), which we currently cannot explain.

The *ADIPOQ* rs182052 is located in intron 1 of the human adiponectin gene (SNP -10069G>A). Although introns are non-protein-coding regions within genes, they are associated with serum adiponectin levels [40]. Many studies have pointed out that the *ADIPOQ* rs182052 A allele is related to diabetes [24,41]. This may be because the *ADIPOQ* rs182052 A allele is related to lower serum adiponectin levels, higher body mass index, higher fasting insulin, thicker skinfold thickness, lower homeostasis model assessment of insulin sensitivity, and diabetic nephropathy [42,43,44]. However, we found the OR of CKD in the *ADIPOQ* rs182052 GG genotype was marginally higher than that of the GA/AA genotype. Whether it is because *ADIPOQ* rs182052 has more GG genotype distribution in CKD cases, and its eGFR is significantly lower than that of controls (Table 3), needs further investigation. Furthermore, we found that the *ADIPOQ* rs182052 GG genotype had significantly or marginally higher OR for CKD than the GA/AA genotype in the group with diabetes or in those with low total urinary arsenic or low blood cadmium concentrations (Table 4). This may be due to the lower adiponectin concentration in the *ADIPOQ* rs182052 GG genotype than in the GA/AA genotype, while at the same time diabetic patients had lower adiponectin, resulting in a higher OR of CKD, which was not observed in non-diabetic patients (normal adiponectin). However, we did not measure adiponectin concentrations; we have no evidence at present. In addition, the effect of the *ADIPOQ* rs182052 gene on CKD was shown at low urinary total arsenic and low blood cadmium concentrations, but at high urinary total arsenic and high blood cadmium concentrations, the effect of the *ADIPOQ* rs182052 gene on CKD could not be presented; it may be that the effect of the *ADIPOQ* rs182052 gene is lower than the effect of metals on CKD, and this needs further investigation. The discrepancy between previous studies and our results may be partly due to different population backgrounds, allelic heterogeneity, and phenotype definitions. However, the genotype frequencies in our control group were similar to those in previous studies [45,46]. Only one study had similar results to ours in which they found a significantly lower risk for ischemic stroke for the *ADIPOQ* rs182052 GA/AA compared to the GG genotype [47]. Furthermore, we found that the *ADIPOQ* rs182052 risk genotype had a significant additive and multiplicative interaction with diabetes to increase the risk of CKD. This may be because diabetes is an important risk factor for CKD, and diabetes is associated with low adiponectin levels [48]. We did not measure serum adiponectin levels to confirm whether the *ADIPOQ* rs182052 GG genotype interacted with diabetes to increase CKD risk through serum adiponectin levels or through other mechanisms, and this requires further exploration.

This study had some limitations. We did not measure adiponectin concentrations, which is one limitation of this study. When assessing the association between *ADIPOQ* polymorphisms and CKD and the interaction of *ADIPOQ* polymorphisms and metal levels on CKD, further studies with larger sample sizes are required to improve the precision of point estimates. The analysis of six *ADIPOQ* polymorphisms could not explain all gene functions. Further functional evaluation of *ADIPOQ* polymorphisms is needed to determine their role in CKD.

## 4. Materials and Methods

### 4.1. Study Subjects

This study recruited 215 CKD patients and 423 age- and sex-matched healthy controls at Taipei Wanfang Medical Center and Taipei Medical University Hospital previously [49]. This study was approved by the Taipei Medical University-Joint Institutional Review Board (N202206001). All study subjects were interviewed using questionnaires and had biological samples collected after providing their informed consent. Nephrologists at Taipei Wanfang Hospital Medical Center and Taipei Medical University Hospital determined the different stages of CKD in patients using serum creatinine, blood urea nitrogen, proteinuria, and the modified diet in renal disease (MDRD) formula to calculate eGFR. The MDRD formula follows eGFR (mL/min/1.73 m^2^) = 186.3 × serum creatinine^−1.154^ × age^−0.203^ × 1.212 (if patient is black) × 0.742 (if female) [50].

### 4.2. Interview and Bio-Specimen Collection

Well-trained interviewers conducted interviews with study subjects using structured questionnaires. The questionnaire included sociodemographic data, use of analgesics, history of diseases, such as hypertension and diabetes, and lifestyle information, such as alcohol, coffee, and tea consumption and cigarette smoking habits.

From each subject, approximately 8 mL of blood was collected using an EDTA vacuum syringe to separate buffy coats for DNA extraction to analyze *ADIPOQ* rs11063539, rs150129, rs182052, rs2241766, rs7627128, and rs822396 genotypes. Plasma was separated for measurement of selenium levels, and red blood cells were separated to measure lead and cadmium concentrations. Spot urine samples were collected and stored in acid-washed tubes and transferred to a −20 °C freezer in preparation for arsenic species analyses.

### 4.3. Arsenic, Cadmium, Lead, and Selenium Measurement

Arsenite (As^III^), arsenate (As^V^), monomethylarsonic acid (MMA^V^), and dimethylarsinic acid (DMA^V^) were separated using high-performance liquid chromatography (Merck Hitachi, Tokyo, Japan). The concentrations of these four species were determined using a hydride generator coupled to an atomic absorption spectrometer (PerkinElmer, Waltham, MA, USA) [51]. Analysis of plasma selenium and blood lead and cadmium levels was performed by inductively coupled plasma mass spectrometry (Agilent Technologies, Santa Clara, CA, USA) [5]. When the experimental value was below the detection limit, half the concentration of the detection limit was used in data analysis. Supplementary Table S1 presents assay methods, detection limits, validity, and reliability for metals or metalloids. Total urinary arsenic concentration was the sum of As^III^, As^V^, MMA^V^, and DMA^V^ concentrations.

### 4.4. Determination of ADIPOQ Gene Polymorphisms

Genomic DNA was obtained by digestion with proteinase K followed by phenol and chloroform extraction. Appropriate single nucleotide polymorphisms (SNPs) were selected based on the frequency of Han Chinese in Beijing, China and Asians from the dbSNP Short Genetic Variations provided by the National Center for Biotechnology Information (https://www.ncbi.nlm.nih.gov/projects/SNP/, accessed on 1 September 2022). The genotypes of *ADIPOQ* rs11063539, rs150129, rs182052, rs2241766, rs7627128, and rs822396 were determined using the Agena Bioscience Mass ARRAY System according to the manufacturer’s instructions.

### 4.5. Statistical Analysis

Medians (interquartile ranges) present continuous variables, and frequencies (percentages) represent categorical variables. The chi-square test was used to test whether the *ADIPOQ* genotypes of the control group fitted Hardy–Weinberg equilibrium and to analyze the distribution of categorical variables among the subject groups. The Wilcoxon rank sum test compared the continuous variables between CKD cases and controls. Multiple logistic regressions were used to estimate ORs and 95% CIs to assess the associations between *ADIPOQ* genotypes and CKD. The association between SNPs for each *ADIPOQ* and CKD was analyzed for trend test using ranked SNPs coded for zero, one, and two minor alleles. The median of urinary total arsenic and blood cadmium concentrations in the control group was used as the cut point for stratification in order to divide all the population into high-concentration group and low-concentration group. The associations between *ADIPOQ* gene polymorphisms and CKD in the high-concentration and low-concentration groups were analyzed, respectively. All models were adjusted for variables such as age, sex, education level, analgesic usage, history of diabetes and hypertension, and alcohol, coffee, and tea consumption. Linkage disequilibrium (LD) strength was determined by calculating Lewontin’s D′ and r^2^ using Haploview 4.1 software [52]. The interaction of metal concentrations (median of control concentrations as cut-off point) and *ADIPOQ* polymorphisms on CKD was also assessed. The linear trend test of the OR of each stratum in the interaction was calculated using the scores of no risk factor, one risk factor, and two risk factors (risk genotype, diabetes, or high metal exposure) as continuous variables. The synergy index was used to analyze the additive interaction of pairwise variables, such as *ADIPOQ* genotype, disease histories, total urinary arsenic, plasma selenium, and blood lead and cadmium concentrations on CKD [53]. The 95% CIs were calculated using the OR and its variance–covariance matrix [54]. Multiplicative interactions between metals, disease history, and *ADIPOQ* polymorphisms on CKD were analyzed using product terms in multiple logistic regression models. Software SAS 9.4 (SAS Institute, Cary, NC, USA) was used to analyze data. Significance was considered with a two-tailed *p* < 0.05.

## 5. Conclusions

We observed a marginal association between *ADIPOQ* rs182052 and CKD. However, to our knowledge, this is the first finding that *ADIPOQ* rs182052 and rs1501299 polymorphisms have respective significant additive and multiplicative interactions with diabetes to enhance the risk of CKD.

## Figures and Tables

**Table 1:** Sociodemographic characteristics, lifestyle, and disease histories for CKD cases and controls.

Variables	CKD Cases (*N* = 215)	Controls (*N* = 423)	Age–Sex Adjusted OR (95% CI)
Age	66.00 (19.00)	66.00 (18.00) ^a^	1.00 (0.99–1.02)
Gender			
Male	132 (61.40)	260 (61.47) ^b^	1.00
Female	83 (38.60)	163 (38.53)	1.02 (0.72–1.44)
Educational level			
Illiterate/elementary school	88 (40.93)	96 (22.70) ^b,^***	1.00
Junior/senior high school	71 (33.02)	146 (34.52)	0.50 (0.33–0.75) **
College and above	56 (26.05)	181 (42.79)	0.31 (0.20–0.48) ***
Cigarette smoking			
Non-smoker	158 (73.49)	307 (72.58) ^b^	1.00
Former smoker	32 (14.88)	73 (17.26)	1.16 (0.66–2.02)
Current smoker	25 (11.63)	43 (10.17)	0.83 (0.51–1.36)
Alcohol consumption			
Never	178 (82.79)	270 (63.83) ^b,^***	1.00
Frequently	33 (15.35)	65 (15.37)	0.71 (0.44–1.14)
Occasional	4 (1.86)	88 (20.80)	0.06 (0.02–0.18) ***
Frequently or occasional	37 (17.21)	153 (36.17)	0.34 (0.22–0.51) ***
Coffee consumption			
Never	167 (77.67)	218 (51.54) ^b,^***	1.00
Frequently	29 (13.49)	102 (24.11)	0.37 (0.23–0.59) ***
Occasional	19 (8.84)	103 (24.35)	0.06 (0.02–0.18) ***
Frequently or occasional	48 (22.33)	205 (48.46)	0.30 (0.21–0.44) ***
Tea consumption			
Never	119 (55.35)	149 (35.22) ^b,^***	1.00
Frequently	70 (32.56)	168 (39.72)	0.52 (0.36–0.75) ***
Occasional	26 (12.09)	106 (25.06)	0.31 (0.19–0.50) ***
Frequently or occasional	96 (44.65)	274 (64.78)	0.43 (0.31–0.61) ***
Analgesic usage			
No/yes as-needed basis	189 (87.91)	404 (95.51) ^b,^***	1.00
Yes, routinely	26 (12.09)	19 (4.49)	2.94 (1.59–5.45) ***
Diabetes			
No	130 (60.47)	379 (89.60) ^b,^***	1.00
Yes	85 (39.53)	44 (10.40)	5.66 (3.73–8.59) ***
Hypertension			
No	93 (43.26)	296 (69.98) ^b,^***	1.00
Yes	122 (56.72)	127 (30.02)	3.17 (2.23–4.50) ***

Abbreviations: CKD, chronic kidney disease; OR, odds ratio; CI, confidence interval; Values are expressed as median (quartile range) or number (%) of cases and controls; ^a^ Wilcoxon rank sum test; ^b^ χ^2^ test; ** *p* < 0.01, *** *p* < 0.001.

**Table 2:** Associations between *ADIPOQ* gene polymorphisms and CKD.

*ADIPOQ* Genotypes	CKD Cases	Controls	Age–Sex Adjusted ORs (95% CI)	Multivariate Adjusted ^a^ ORs (95% CI)
rs182052 G>A				
AA	33 (15.64)	76 (18.27)	1.00	1.00 ^§,+^
GA	103 (48.81)	214 (51.44)	1.11 (0.69–1.78)	1.14 (0.65–2.00)
GG	75 (35.55)	126 (30.29)	1.38 (0.84–2.27)	1.63 (0.90–2.97)
GG vs. GA/AA			1.27 (0.90–1.81)	1.48 (0.97–2.26)^+^
rs1501299 G>T				
GG	106 (49.77)	230 (54.89)	1.00	1.00
GT	95 (44.60)	152 (36.28)	1.36 (0.97–1.93) ^+^	1.19 (0.79–1.79)
TT	12 (5.63)	37 (8.83)	0.71 (0.36–1.42)	0.73 (0.33–1.60)
GT/TT	107 (50.23)	189 (45.11)	1.23 (0.89–1.72)	1.20 (0.82–1.74)
rs1063539 G>C				
GG	104 (48.60)	198 (48.06)	1.00	1.00
GC	91 (42.52)	176 (42.72)	0.99 (0.70–1.40)	0.89 (0.59–1.35)
CC	19 (8.88)	38 (9.22)	0.96 (0.53–1.75)	0.94 (0.47–1.90)
rs2241766 G>T				
GG	27 (12.62)	56 (13.59)	1.00	1.00
GT	99 (46.26)	165 (40.05)	1.17 (0.64–2.15)	1.13 (0.56–2.29)
TT	88 (41.12)	191 (46.36)	0.93 (0.51–1.70)	0.97 (0.48–1.95)
rs7627128 A>C				
AA	8 (3.77)	19 (4.52)	1.00	1.00
AC	66 (31.13)	156 (37.14)	1.01 (0.42–2.43)	0.58 (0.20–1.67)
CC	138 (65.09)	245 (58.33)	1.35 (0.58–3.17)	0.93 (0.33–2.59)
rs822396 A>G				
AA	164 (77.73)	335 (80.34)	1.00	1.00
AG	47 (22.27)	75 (17.99)	1.30 (0.86–1.96)	1.18 (0.72–1.93)
GG	0 (0.00)	7 (1.68)		

Abbreviations: CKD, chronic kidney disease; *ADIPOQ*, adiponectin; OR, odds ratio; CI, confidence interval. Twelve participants were missing for *ADIPOQ* rs11063539, three were missing for *ADIPOQ* rs150129, eleven were missing for *ADIPOQ* rs182052, twelve were missing for *ADIPOQ* rs2241766, six were missing for *ADIPOQ* rs7627128, and ten were missing for *ADIPOQ* rs822396. ^a^ Adjusted for age, sex, educational level, analgesic usage, disease histories of diabetes and hypertension, and alcohol, coffee, and tea consumption. ^§^ Trend test; ^+^ 0.05 < *p* < 0.1.

**Table 3:** Serum creatinine, total urinary arsenic, blood cadmium and lead, and plasma selenium concentrations and eGFR for CKD cases and controls.

	CKD Cases	Controls	
Variables	Median (Quartile Range)	Median (Quartile Range)	*p*
Serum creatinine (mg/dL)	2.00 (1.70)	0.90 (0.20)	<0.0001
eGFR (mL/min/1.73 m^2^)	32.24 (25.76)	80.83 (19.70)	<0.0001
Total urinary arsenic (μg/g creatinine)	22.53 (18.93)	16.04 (15.36)	<0.0001
Blood cadmium (μg/L)	1.66 (1.47)	1.04 (0.82)	<0.0001
Blood lead (μg/dL)	63.72 (46.71)	42.29 (27.08)	<0.0001
Plasma selenium (μg/L)	185.68 (75.65)	217.85 (70.55)	<0.0001

Quartile range: third quartile–first quartile; eGFR, estimated glomerular filtration rate; *p*-value tested by Wilcoxon rank-sum test.

**Table 4:** Associations between *ADIPOQ* gene polymorphisms and CKD stratified by diabetes and total urinary arsenic and blood cadmium levels.

Diabetes				Non-Diabetes		
*ADIPOQ* Genotypes	CKD Cases/ Controls	Age–Sex Adjusted ORs (95% CI)	Multivariate Adjusted ^a^ ORs (95% CI)	CKD Cases/ Controls	Age–Sex Adjusted ORs (95% CI)	Multivariate Adjusted ^a^ ORs (95% CI)
rs182052 G>A						
AA	14/10	1.00 ^§,^*	1.00 ^§,^*	19/66	1.00	1.00
GA	38/28	0.97 (0.37–2.51)	0.75 (0.24–2.32)	65/186	1.20 (0.67–2.16)	1.26 (0.66–2.41)
GG	32/5	4.51 (1.29–15.73) *	4.48 (1.02–19.66) *	43/121	1.22 (0.66–2.28)	1.33 (0.67–2.65)
GG vs.GA/AA		4.62 (1.64–13.01) **	5.54 (1.64–18.74) **		1.06 (0.69–1.63)	1.11 (0.69–1.80)
rs1501299 G>T						
GG	44/15	1.00 ^§,^*	1.00 ^§,^*	62/215	1.00	1.00
GT	36/20	0.62 (0.28–1.38)	0.40 (0.15–1.06) ^+^	59/132	1.55 (1.02–2.36) *	1.55 (0.97–2.46) ^+^
TT	4/8	0.16 (0.04–0.62) **	0.27 (0.05–1.39)	8/29	0.96 (0.42–2.22)	1.05 (0.42–2.58)
GT/TT	40/28	0.49 (0.23–1.04) ^+^	0.37 (0.15–0.96) *	67/161	1.45 (0.97–2.16) ^+^	1.46 (0.94–2.27) ^+^
	Total urinary arsenic ≤ 16.04 μg/g creatinine			Total urinary arsenic > 16.04 μg/g creatinine		
rs182052 G>A						
AA	5/38	1.00 ^§,^*	1.00 ^§,^*	28/37	1.00	1.00
GA	28/105	1.84 (0.65–5.16)	2.49 (0.77–7.99)	75/108	0.89 (0.50–1.59)	0.80 (0.40–1.30)
GG	24/61	3.05 (1.06–8.78) *	4.06 (1.22–13.57) *	51/65	1.05 (0.56–1.94)	1.20 (0.54–2.67)
GG vs. GA/AA		1.88 (1.01–3.50) *	1.96 (0.97–3.94) ^+^		1.14 (0.73–1.79)	1.39 (0.78–2.50)
	Blood cadmium ≤ 1.04 μg/L			Blood cadmium > 1.04 μg/L		
rs182052 G>A						
AA	8/39	1.00	1.00	25/37	1.00	1.00
GA	19/108	0.81 (0.32–2.01)	0.78 (0.26–2.52)	84/106	1.15 (0.64–2.06)	1.09 (0.54–2.23)
GG	18/64	1.28 (0.50–3.26)	1.91 (0.61–5.99)	57/62	1.31 (0.70–2.46)	1.29 (0.60–2.78)
GA/AA		1.50 (0.77–2.92)	2.27 (0.95–5.43) ^+^		1.18 (0.76–1.83)	1.20 (0.71–2.04)

Abbreviations: *ADIPOQ*, adiponectin; OR, odds ratio; CI, confidence interval. ^a^ Adjusted for age, sex, educational level, analgesic usage, disease histories of diabetes and hypertension, and alcohol, coffee, and tea consumption; ^§^ Trend test, ^+^ 0.05 < *p* <0.1, * *p* < 0.05, ** *p* < 0.01; Two participants were missing for *ADIPOQ* rs150129 in diabetes and four missing for *ADIPOQ* rs150129 in non-diabetes; two were missing for *ADIPOQ* rs182052 in diabetes and nine missing for *ADIPOQ* rs182052 in non-diabetes; two were missing in total urinary arsenic ≤ 16.04 μg/g creatinine and five missing in total urinary arsenic > 16.04 μg/g creatinine for *ADIPOQ* rs182052; and six were missing in blood cadmium ≤ 1.04 μg/L and five in blood cadmium ≤ 1.04 μg/L for *ADIPOQ* rs182052.

**Table 5:** Pairwise analyses of combined effects of diabetes, total urinary arsenic level, blood cadmium level, and *ADIPOQ* risk genotype on CKD.

Risk Factor	Risk Factor	Case/Control	Age–Sex Adjusted ORs (95% CI)	Multivariate Adjusted ORs (95% CI) ^a^
*ADIPOQ* rs182052	Diabetes			
GA/AA No	No	84/252	1.00 ^§,^***	1.00 ^§,^***
GG	No	43/121	1.07 (0.70–1.64)	1.12 (0.69–1.81)
GA/AA	Yes	52/38	4.11 (2.52–6.69) ***	3.19 (1.83–5.54) ***
GG	Yes	32/5	19.21 (7.25–50.89) ***	16.33 (5.72–46.66) ***
		Synergy index	5.73 (1.82–18.10)	6.64 (1.81–24.36)
		*p* _interaction_	0.010	0.015
*ADIPOQ* rs1501299	Diabetes			
GG	No	62/215	1.00 ^§,^***	1.00 ^§,^***
GT/TT	No	67/161	1.45 (0.97–2.17) ^+^	1.47 (0.94–2.30) ^+^
GG	Yes	44/15	10.22 (5.32–19.60) ***	9.24 (4.44–19.21) ***
GT/TT	Yes	40/28	4.96 (2.83–8.70) ***	3.69 (1.98–6.87) ***
		Synergy index	0.41 (0.17–0.98)	0.31 (0.11–0.86)
		*p* _interaction_	0.013	0.001
*ADIPOQ* rs182052	Total urinary arsenic (μg/g creatinine)			
GA/AA No	<16.04	33/145	1.00 ^§,^***	1.00 ^§,^***
GG	<16.04	24/61	(0.94–3.18) ^+^	2.07 (1.04–4.13) *
GA/AA	>16.04	103/145	3.16 (2.00–5.00) ***	2.79 (1.64–4.77) ***
GG	>16.04	51/65	3.46 (2.04–5.87) ***	3.59 (1.90–6.79) ***
		Synergy index	0.85 (0.44–1.65)	0.91 (0.40–2.05)
		*p* _interaction_	0.531	0.913
*ADIPOQ* rs182052	Blood cadmium (μg/L)			
GA/AA No	<1.04	27/147	1.00 ^§,^***	1.00 ^§,^***
GG	<1.04	18/64	1.52 (0.78–2.96)	2.14 (0.96–4.77) ^+^
GA/AA	>1.04	109/143	4.33 (2.66–7.06) ***	5.26 (2.90–9.53) ***
GG	>1.04	57/62	5.16 (2.98–8.94) ***	6.25 (3.21–12.17) ***
		Synergy index	1.08 (0.61–1.93)	0.97 (0.50–1.87)
		*p* _interaction_	0.441	0.259

Abbreviations: *ADIPOQ*, adiponectin; OR, odds ratio; CI, confidence interval. ^a^ Adjusted for age, sex, educational level, analgesic usage, disease histories of diabetes and hypertension, and alcohol, coffee, and tea consumption. ^§^ Trend test; ^+^ 0.05 < *p* < 0.1, * *p* < 0.05, *** *p* < 0.001; *p* _interaction_: multiplicative interaction. Three were missing for *ADIPOQ* rs150129 and eleven missing for *ADIPOQ* rs182052. Eleven were missing in the combined effect for total urinary arsenic and *ADIPOQ* rs182052, eleven missing in the combined effect of blood cadmium and *ADIPOQ* rs182052; and twelve missing in the combined effect of blood cadmium and *ADIPOQ* rs1063539.

## Data Availability

The data presented in this study are available on request from the corresponding author. The data are not publicly available due to privacy or ethical restrictions.

## Supplementary Materials

The following supporting information can be downloaded at: https://www.mdpi.com/article/10.3390/ijms24098128/s1.
