# Supplementary material for: Chronic Kidney Disease: Interaction of Adiponectin Gene Polymorphisms and Diabetes
Source: Int J Mol Sci. 2023 May 1;24(9):8128. doi: 10.3390/ijms24098128 (PMC10179290; doi:10.3390/ijms24098128)
Supplement: Supplementary file 1 [file ijms-24-08128-s001.zip › ijms-2365825-supplementary.pdf]

Supplementary Table

Supplementary Table S1. Validity and reliability of urinary arsenic species, plasma selenium, and red blood cell lead and cadmium.

Metals or metalloids	Method	Detection limit (μg/L)	Recovery rate	SRM	CV
Plasma selenium	Inductively coupled plasma mass spectrometry	0.193		SRMs (Seronorm Trace Elements Whole Blood L-2; SERO AS, Norway) contained 112 ± 46 mg/L of selenium, in our system 118.7 ± 11.1 mg/L (n = 7)	9.8%
Red blood cell lead	Inductively coupled plasma mass spectrometry	0.32		SRMs [Seronorm Trace Elements Whole Blood L-2 (Lot 1103129)] certificate value 310.0 μg/L (range 186.0–434.0 μg/L), in our system 329.0 ± 17.0 μg/L	<10%
Red blood cell cadmium	Inductively coupled plasma mass spectrometry	0.07		SRMs [Seronorm Trace Elements Whole Blood L-2 (Lot 1103129)] certificate value 5.8 μg/L (range: 5.4–6.2 μg/L), in our system 6.1 ± 0.5 μg/L	<10%
Arsenite (As^{III})	High-performance liquid chromatography–hydride generator–atomic absorption spectrometry	0.02	93.8–102.2%	SRM (National Institute of Standards and Technology, NIST; Gaithersburg, MD) 2670 certificate value 480 ± 100 μg/L inorganic arsenic, in our system 507 ± 17 μg/L (n = 4)	<10%
Arsenate (As^{V})		0.10			
Monomethylarsonic acid (MMA^{V})		0.07			
Dimethylarsinic acid (DMA^{V})		0.06			

(A) (B)

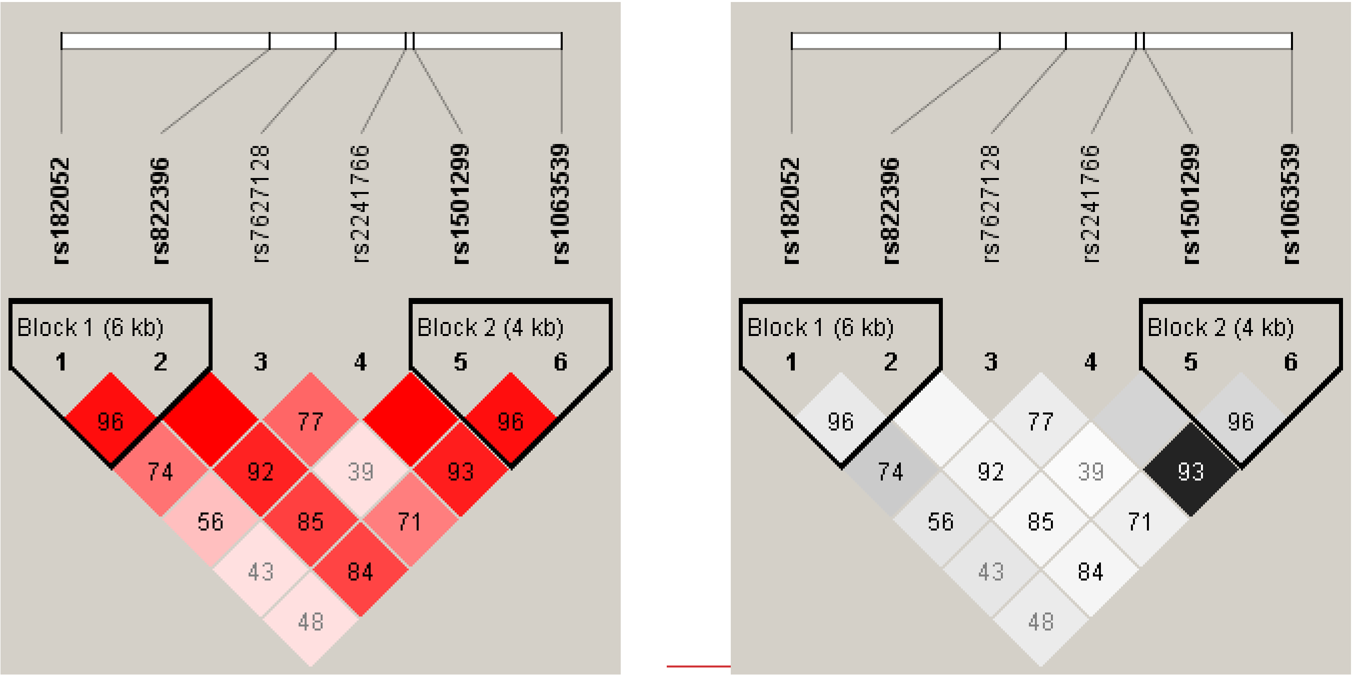

Figure S1. (A) Lewontin's D′ of *ADIPOQ* block 1 (*ADIPOQ* rs182052 and *ADIPOQ* rs822396), *ADIPOQ* block 2 (*ADIPOQ* rs1501299 and *ADIPOQ* rs1063539) polymorphisms. (B) r^2 values for each pair of polymorphisms of *ADIPOQ*.

Supplementary Table S2. Haplotypes of *ADIPOQ* genes and the risk of CKD

Haplotypes of *ADIPOQ*	CKD cases (N= 218)	Controls (N = 427)	Age-sex adjusted ORs (95% CI)	Multivariate adjusted ORs (95% CI)[a]
ADIPOQ block 1: *ADIPOQ* rs182052 and rs822396				
G-A	206 (49.28)	372 (45.59)	1.00	1.00
A- A	166 (39.71)	357 (43.75)	0.84 (0.65−1.08)	0.78 (0.57−1.05)[+]
G-G or A-G	46 (11.01)	87 (10.66)	0.95 (0.64−1.41)	0.90 (0.56−1.45)
ADIPOQ block 2: *ADIPOQ* rs1501299 and rs1063539				
G-G	175 (41.87)	346 (42.40)	1.00	1.00
G-C	126 (30.14)	246 (30.15)	1.02 (0.77−1.35)	0.94 (0.67−1.31)
T-G or T-C	117 (27.99)	224 (27.45)	1.04 (0.78−1.39)	0.96 (0.68−1.36)

Abbreviations: *ADIPOQ*, adiponectin; OR, odds ratio; CI, confidence interval.
In haplotype blocks 1 and 2, there are 18 alleles missing in cases and 38 alleles missing in controls.
[a] Adjusted for age, gender, educational level, analgesic usage, disease histories of diabetes and hypertension, and alcohol, coffee, and tea consumption.
[+] $0.05 \leq p < 0.1$.
